# Supplementary material for: Utilizing Dynamic Contrast-Enhanced Magnetic Resonance Imaging (DCE-MRI) to Analyze Interstitial Fluid Flow and Transport in Glioblastoma and the Surrounding Parenchyma in Human Patients
Source: Pharmaceutics. 2021 Feb 4;13(2):212. doi: 10.3390/pharmaceutics13020212 (PMC7913790; doi:10.3390/pharmaceutics13020212)
Supplement: Supplementary file 1 [file pharmaceutics-13-00212-s001.pdf]

pharmaceutics

Supplementary Materials: Utilizing Dynamic Contrast-Enhanced Magnetic Resonance Imaging (DCE-MRI) to Analyze Interstitial Fluid Flow and Transport in Glioblastoma and the Surrounding Parenchyma in Human Patients

Krishnashis Chatterjee [1,†], Naciye Atay [1,†], Daniel Abler [2,3], Saloni Bhargava [1], Prativa Sahoo [2], Russell C. Rockne [2] and Jennifer M. Munson [1,*]

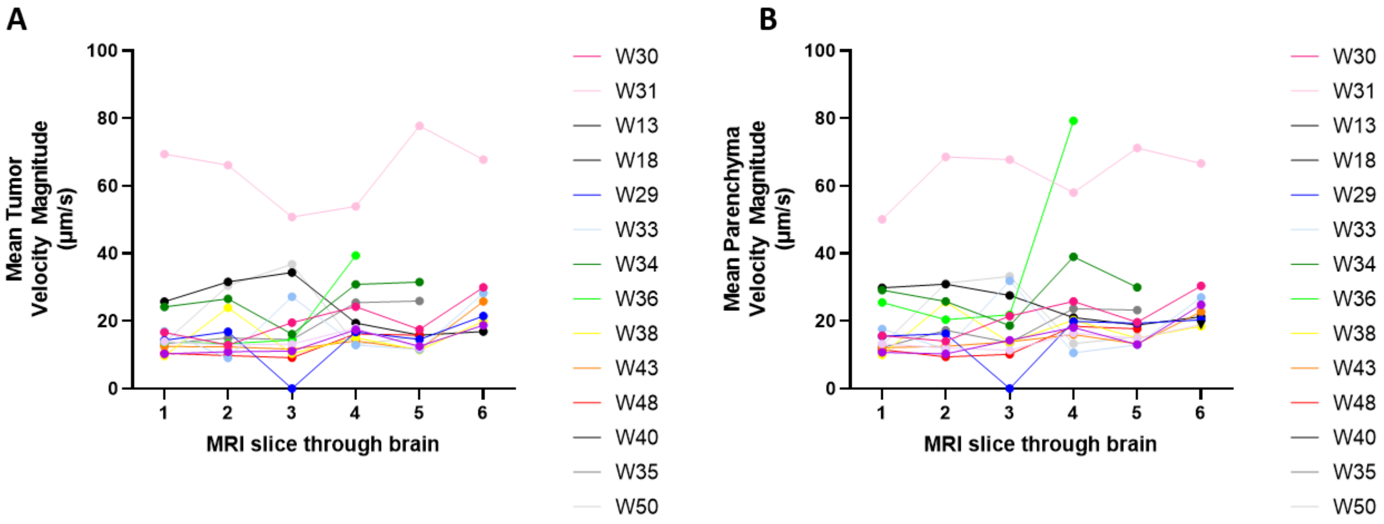

Figure S1. Mean tumor velocity magnitude by slice through brain. A) On a patient-by-patient basis, the total velocity magnitude in the tumor was averaged per slice, and **B)** similar analysis in the parenchymal surrounding space. Each color indicates a unique patient and is consistent with the figures in the main manuscript, as well as Table S1.

Publisher's Note: MDPI stays neutral with regard to jurisdictional claims in published maps and institutional affiliations.

Copyright: © 2020 by the authors. Submitted for possible open access publication under the terms and conditions of the Creative Commons Attribution (CC BY) license (http://creativecommons.org/licenses/by/4.0/).

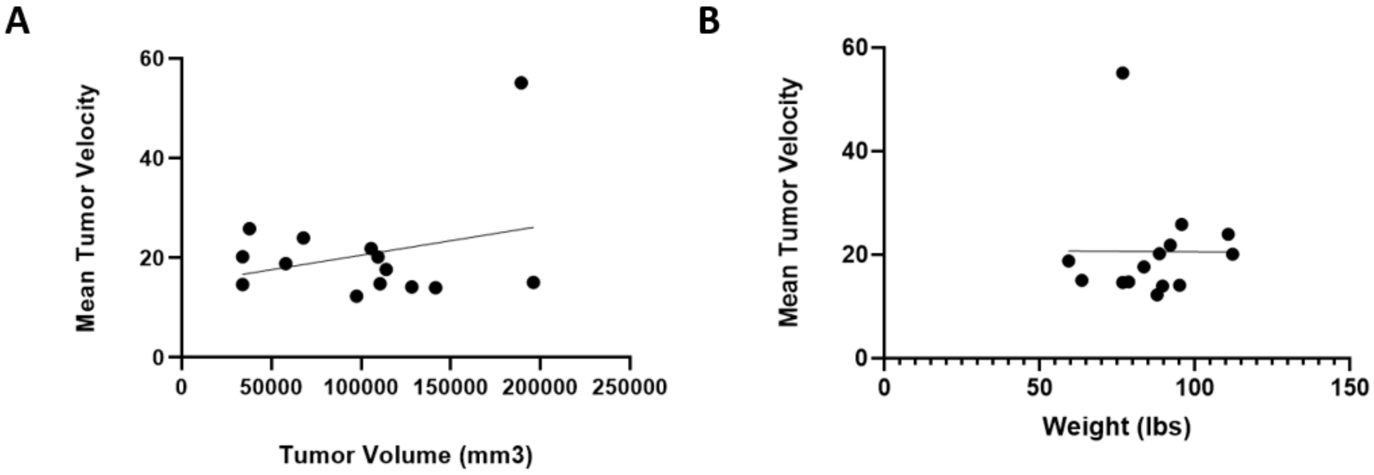

C

Comparison Variable	P-value
Sex	0.1469
MGMT Methylation Status	0.9497
EGFR Amplification	0.329

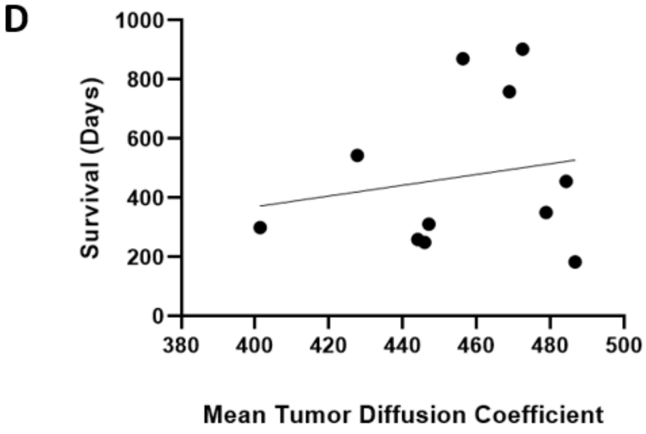

Figure S2. Patient parameters and outcomes on IFF and diffusion. (A) Mean tumor velocity vs. calculated tumor volume from 6 slices on MRI. (B) Mean tumor velocity vs patient weight at MRI. (C) Table of Mann-Whitney t-test comparisons for three reported binary (as reported) variables. (D) Correlation of survival with mean tumor diffusion coefficient.

Table S1. Select patient information from the Ivy GAP Database This table includes patient specific clinical data from the Ivy GAP database for the patients analyzed in this study. More detailed information is available for each patient in the database for registered users [1].

Patient No.	Histopathology	Molecular Subtype	Gender	Initial Diagnosis Age (years)	KPS	Weight (kg)	Height (cm)	Location of Tumor	Surgery	Extent of Resection	Surgery (days since initial diagnosis)	Chemotherapy	Radiation Therapy	1p19q Deletion	EGFR	PTEN	MGMT PCR	IDH1	EGFR vIII	Time to Progression (days)	Survival (days)	Cause of Death
W13-1-1	Glioblastoma	Mesenchymal	Female	60	90	95	160	Right Parietal	Primary	Complete resection	6	Yes	Yes	Negative	Amplification	Loss	Unmethylated	Wildtype	Yes	235	250	Deceased due to tumor progression
W18-1-1	Glioblastoma, giant cell type, WHO grade IV	-	Female	37	90	78.654	173.72	Right Parietal	Primary	Subtotal resection 90%	4	Yes	Yes	Negative	Gain	Gain	Methylated	Wildtype	No	489	903	Deceased due to tumor progression
W29-1-1	Glioblastoma	Classical, Neural	Male	74	100	89.54	179	Right Parietal	Primary	Complete resection	2	Yes	Yes	Negative	Amplification	Deletion	Unmethylated	Wildtype	Yes	246	260	Deceased due to tumor progression
W30-1-1	Glioblastoma, WHO grade IV	-	Male	60	70	112.1	-	Left Parietal	Primary	Subtotal resection 90%	4	Yes	Yes	Negative	Amplification	Deletion	Methylated	Wildtype	No	-	759	Deceased due to tumor progression
W31-1-1	Glioblastoma, WHO grade IV	Proneural	Male	18	90	76.7	179	Left Frontal	Primary	Complete resection	1	Yes	Yes	Positive	Gain	Deletion	Unmethylated	R132H	No	312	871	Deceased due to tumor progression
W33-1-1	Glioblastoma, WHO grade IV	Classical	Male	61	80	83.5	183	Right Parietal	Primary	Complete resection	3	Yes	Yes	Negative	Amplification	Loss	Methylated	Wildtype	No	-	-	-
W34-1-1	Glioblastoma, WHO grade IV	Classical, Mesenchymal	Male	74	90	95.7	185	Left Temporal	Primary	Complete resection	1	Yes	Yes	Negative	Gain	Deletion	Unmethylated	Wildtype	No	170	351	Deceased due to tumor progression

W35-1-1	Anaplastic astrocytoma WHO grade III/IV	-	Female	37	100	59.3	169	Right Parietal	Primary	Complete resection	7	Yes	Yes	Negative	Gain	Gain	Methylated	R132H	No	-	-	-
W36-1-1	Glioblastoma, WHO grade IV	Mesenchymal	Male	62	80	88.5	178	Left Parietal	Primary	Subtotal resection 90%	3	Yes	Yes	Negative	Gain	Gain	Unmethylated	Wildtype	No	241	544	Deceased due to tumor progression
W38-1-1	Glioblastoma, WHO grade IV/IV	Proneural	Female	65	70	63.5	170.2	Right Temporal	Primary	Subtotal resection 90%	5	Yes	Yes	Negative	Gain	Normal	Methylated	Wildtype	No	236	311	Deceased due to tumor progression
W40-1-1	Glioblastoma	Mesenchymal, Neural	Male	65	100	110.7	172.7	Left Parietal	Primary	Complete resection	3	Yes	Yes	Negative	Gain	Loss	Unmethylated	Wildtype	No	-	184	Deceased due to tumor progression
W43-1-1	Glioblastoma	Neural	Female	62	90	76.749	163.56	Right Temporal	Primary	Complete resection	1	Yes	Yes	-	-	-	-	Wildtype	-	133	300	Deceased due to tumor progression
W48-1-1	Glioblastoma	-	Male	52	80	87.8	177.8	Right Parietal	Primary	Complete resection	1	Yes	Yes	-	-	-	-	R132G	No	92	456	Deceased due to tumor progression
W50-1-1	Glioblastoma WHO grade IV	-	Male	27	90	92.08	185.4	Left Parietal	Primary	Complete resection	6	Yes	Yes	-	-	-	-	Wildtype	No	93	-	-

References

1. Shah, N.; Feng, X; Lankerovich, M; Puchalski, R.B.; Keogh, B. Data from Ivy GAP. The Cancer Imaging Archive. **2016**. http://dx.doi.org/10.7937/K9/TCIA.2016.XLwaN6nL
